# Concurrent participation in screening for cervical, breast, and bowel cancer in England

**DOI:** 10.1177/0969141319871977

**Published:** 2019-09-16

**Authors:** Matejka Rebolj, Dharmishta Parmar, Roberta Maroni, Oleg Blyuss, Stephen W Duffy

**Affiliations:** 1 Cancer Prevention Group, School of Cancer & Pharmaceutical Sciences, Faculty of Life Sciences & Medicine, King’s College London, London, UK; 2 Centre for Cancer Prevention, Wolfson Institute of Preventive Medicine, Barts & The London School of Medicine and Dentistry, Queen Mary University of London, London, UK; 3 Department of Paediatrics, Sechenov University, Moscow, Russia

**Keywords:** Breast cancer, cervical cancer, bowel cancer, screening, participation

## Abstract

**Objectives:**

To determine how many women participate in all three recommended cancer screening programmes (breast, cervical, and bowel). During their early 60s, English women receive an invitation from all the three programmes.

**Methods:**

For 3060 women aged 60–65 included in an England-wide breast screening case–control study, we investigated the number of screening programmes they participated in during the last invitation round. Additionally, using the Fingertips database curated by Public Health England, we explored area-level correlations between participation in the three cancer screening programmes and various population characteristics for all 7014 English general practices with complete data.

**Results:**

Of the 3060 women, 1086 (35%) participated in all three programmes, 1142 (37%) in two, 526 (17%) in one, and 306 (10%) in none. Participation in all three did not appear to be a random event (*p* < 0.001). General practices from areas with less deprivation, with more patients who are carers or have chronic illnesses themselves, and with more patients satisfied with the provided service were significantly more likely to attain high coverage rates in all programmes.

**Conclusions:**

Only a minority of English women is concurrently protected through all recommended cancer screening programmes. Future studies should consider why most women participate in some but not all recommended screening.

## Introduction

About 50% of British men and 40% of women born in 1950 or later will develop (any) cancer during their life,^1^ and about half will die within 10 years after the diagnosis.^2^ Life-saving screening for cervical, breast, and bowel cancer is currently offered in many developed countries. While cytological screening prevents substantially more than half of all deaths due to cervical cancer,^3^ mammography prevents about 25% of deaths due to breast cancer among those who are invited to screening.^4^ Faecal occult blood testing can prevent about 15% of deaths due to bowel cancer, although other methods such as flexible sigmoidoscopy can potentially prevent even more.^5,6^ Women tend to be invited for screening for multiple cancers concurrently throughout a large part of their adult lives: for cervical screening every three to five years, for breast screening every two to three years, and for bowel screening every two years.^7^ They may receive as many as six to eight screening invitations every five years during their 50s and 60s. Most welcome the idea of having cancer screening available to them, even if they do not invariably take up the offer.^8^ This is reflected in relatively high observed screening participation, although participation varies by country and cancer type.^7,9^

Factors associated with non-participation are largely shared between the three screening programmes.^10–15^ Generally, non-participants are less likely than participants to be aware of screening, and are also less likely to exhibit behaviours consistent with a healthy lifestyle.^16–19^ Based on these shared patterns, it might be hypothesized that the three screening programmes must reach roughly the same women. Multiple studies showing that the likelihood of participation in a given screening programme increases with participation in a different programme could be considered to support this hypothesis.^20–26^ In the case of English women in their 60s, where the lowest participation is observed for bowel screening (57%),^27^ this would mean that close to half of all women should be simultaneously protected from an early death from all three cancers. This, however, has not been tested previously, and doing so is the aim of our study.

## Methods

In England, cancer screening is implemented by the National Health Service (NHS) free of charge. Cervical screening with cytology started in 1988. The programme invites women aged 25–64, with women above age 50 recommended five-yearly screening (younger women are invited every three years). Mammographic breast screening also started in 1988, targeting women aged 50–64 (50–70 since 2005) every three years. Bowel screening with guaiac faecal occult blood testing started in 2006, although it was not fully rolled out until 2010. It invites men and women aged 60–69 (60–74 since 2010) every two years. No other type of cancer screening is currently offered to the English population. Screening invitations are sent by each programme separately, and are not timed to coincide even if several are sent in the same calendar year. Officially reported five-year coverage rates in cervical screening for women aged 60–64 with a cervix were 75% in 2010 and 73% in 2011.^28^ Of all resident women attaining that age, 21% had had a hysterectomy,^29^ which means that 58–59% ([75% or 73%] × (100%–21%)) of all resident women had been screened. In breast screening, three-year coverage at age 60–64 was 78% in 2010/11,^30^ while bowel screening uptake among women invited for the first time was estimated to be 57% in 2010 and 59% in 2011 (coverage rates have not been reported).^27^

### Individual-level data

The individual-level data were from women who served as controls in a nationwide case–control study on the English breast screening programme,^31^ which evaluates the effect of breast screening on breast cancer mortality. Cases died from breast cancer aged 47–89 in 2010–2011 (the most recent data made available for this research) and were diagnosed at age 47–89 in 1990 or later. Two controls were matched on age and screening area to each case (relaxed to a single control if necessary). They were alive at their matched case’s date of death, and were selected from among all women registered with the English NHS since at least age 47. The matched case’s date of death was the last date on which the controls were known to be alive. We refer to this as the woman’s reference date, and her screening participation was determined prior to this date. National Health Application and Infrastructure Services at NHS Digital extracted screening records for each woman from the inception of the three programmes.

**Figure 1.**
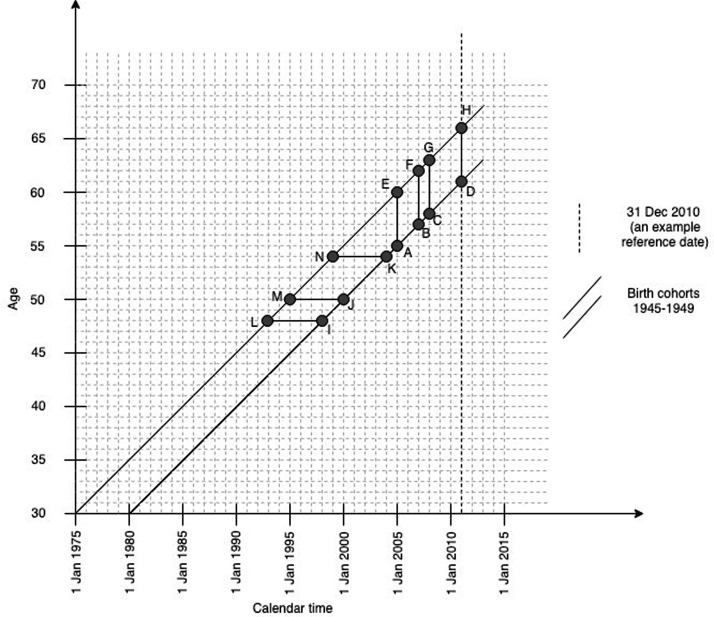
Study design for individual-level data. Main analysis, example for women last known to be alive (=reference date) on 31 December 2010. ADHE: the six-year period to determine participation in cervical screening. BDHF: the four-year period to determine participation in breast screening. CDHG: the three-year period to determine participation in bowel screening. Additional analysis, to determine participation in breast and cervical screening before age 54 years. LIKN: the six-year period to determine participation in cervical screening. MJKN: the four-year period to determine participation in breast screening.

Women were “concurrently” screened in the last round (before the reference date) if they had at least one screening record from each programme during the recommended interval plus one year (to account for reasonable delays), in six years for cervical (ADHE in Figure 1), four years for breast (BDHF), and three years for bowel screening (CDHG). Women must have been aged 60–65 at their reference date. Younger women are not invited for bowel screening, whereas older women may not have been eligible for cervical screening in the last six years. For a reference date in 2010, those who satisfied this age criterion were born in 1945–1949. For a reference date in 2011, this was 1946–1950. The youngest women in these cohorts turned 60 in the calendar year preceding the reference date. From the 17,993 controls in the complete case–control study, 3060 (17%) were born in the selected years.

We categorized women according to their concurrent participation in the last screening round: in all three programmes, in none, or in any combination of two or a single programme. We tested the assumption that concurrent (non-)participation was randomly distributed by calculating the χ^2^ statistic comparing the observed with the expected numbers of women who participated in the last round in all three programmes, or in none. To determine the expected numbers, we assumed that some women consistently avoid participating in any screening, and divided the studied population into women who underwent screening at least once in at least one programme (ever participants, N_EP_) and those who were never screened in any programme (never participants, N_NP_) according to screening records since the inception of the programmes. The expected number of women concurrently participating in all three programmes under the assumption of randomness was:

$${\text{N}}_{\text{EP}}\times \left({\text{N}}_{\text{breast}}/{\text{N}}_{\text{EP}}\right)\times \left({\text{N}}_{\text{cervix}}/{\text{N}}_{\text{EP}}\right)\times \left({\text{N}}_{\text{bowel}}/{\text{N}}_{\text{EP}}\right)+{\text{N}}_{\text{NP}}\times 0$$

where N_breast_, N_cervix_, and N_bowel_ denoted women who participated in the last round before the reference date in breast, cervical, and bowel screening, respectively. Accordingly, the expected number of women not participating in the last round in any programme was:

$${\text{N}}_{\text{EP}}\times \left(\left({\text{N}}_{\text{EP}}-{\text{N}}_{\text{breast}}\right)/{\text{N}}_{\text{EP}}\right)\times \left(\left({\text{N}}_{\text{EP}}-{\text{N}}_{\text{cervix}}\right)/{\text{N}}_{\text{EP}}\right)\times \left(\left({\text{N}}_{\text{EP}}-{\text{N}}_{\text{bowel}}\right)/{\text{N}}_{\text{EP}}\right)+{\text{N}}_{\text{NP}}$$

For women participating in a given programme, we reported the proportions who also participated in no, one, or both other programmes. The same was done for women not participating in the same programme. We compared the patterns by calculating relative proportions (RPs) and calculated the 95% confidence intervals (CIs) for RPs by assuming that their logarithms were approximately normally distributed.

We tested the robustness of the results on two aspects. Firstly, we did not have information on the women’s hysterectomy status. The proportion of women without a cervix increases with age; in 2010–2011, 6% of English women aged 45–49 and 21% of those aged 60–64 ceased to be eligible for cervical screening after a hysterectomy.^29^ Including the same birth cohorts as in the main analysis, we assessed concurrent screening participation before age 54, i.e. when women were at their youngest to be eligible for two screening programmes. Here, concurrent participation was determined as at least one cervical screening sample in the six-year period (LIKN in Figure 1), combined with at least one breast screening sample in the four-year period preceding the 54th birthday (MJKN). Secondly, our data originate from a period when bowel screening was in the process of being rolled out nationally. We repeated the main analysis by restricting the studied sample to 2391 (78%) women with a record of bowel screening invitation sent in the year preceding their reference date at the latest. Here, participation in screening was determined within the same time windows as in the main analysis (Figure 1: ADHE for cervical, BDHF for breast, and CDHG for bowel screening).

### General practice data

Because no information on the women’s background was available from the case–control dataset, we used general practice data from the same source population to investigate area-level correlations between screening participation and various population characteristics. The Fingertips database curated by Public Health England reports health-related data for England, aggregated by administrative area. It was accessed through fingertipsR package for R version 0.2.0.^32^ We retrieved the most recent data (financial year 2017/2018, when all programmes were rolled out nationally), and selected all 7099 general practices with a known Quality of Outcomes Framework list size. We excluded 85 (1.2%) practices with any missing data on practice’s population characteristics or screening participation, leaving 7014 practices for the analysis. Fingertips aggregates screening participation by general practice for each programme separately, and reports it as: the proportion of women aged 50–70 screened for breast cancer in the prior 36 months; the proportion of women aged 25–64 screened for cervical cancer in prior 3 or 5 years (depending on the woman’s age); and the proportion of persons aged 60–69 screened for bowel cancer in the prior 30 months (see Supplemental Appendix). The available practice’s population characteristics included those related to (a) the burden of chronic conditions (proxies: proportion of patients with long-standing health conditions, and the proportion with caring responsibilities other than for underage children), (b) deprivation (proxies: index of multiple deprivation (IMD), proportion of unemployed patients), (c) health behaviours (proxy: proportion of patients smoking), and (d) satisfaction with the general practice (proxy: proportion of patients that would recommend the general practice to others). IMD is a standard English composite measure of deprivation taking into account employment rate, income, education, health, crime, housing, and living environment within a postcode.^33^ None of the definitions of the screening and descriptive indicators could be manipulated, e.g. broken down into narrower age and sex groups, by users.

We defined three groups of practices: those with the same or higher, those with a lower screening coverage than the national average for all three programmes concurrently (“high-coverage practices” and “low-coverage practices”, respectively), and those where the coverage was higher in some but lower in other programmes (“mixed-coverage practices”). We implicitly assumed that the high-coverage practices were more likely to include women who participated in all three programmes concurrently, although we could not verify this independently. We categorized practice characteristics into approximately tertiles (in as much as possible after rounding on the second decimal). We used logistic regression to calculate odds ratios (OR) for high-coverage or mixed-coverage separately vs. low-coverage, depending on practice characteristics. For each practice characteristic, the OR was adjusted for all other practice characteristics under study.

Analyses were undertaken with RStudio, version 1.1.463 (R version 3.5.2).^34^

## Results

### Individual-level data

Of the 3060 women, most (2989, N_EP_; 98%) had at least one screening participation record ever, and 71 (N_NP_; 2%) were never screened in any of the programmes. In the last screening round before the reference date, 2525 (N_breast_; 83%) women were screened for breast, 1908 (N_cervix_; 62%) for cervical, and 1635 (N_bowel_; 53%) for bowel cancer, which is consistent with the proportions reported in the official statistics for England (78%, 58–59%, and 57–59%, respectively, as reported above).

**Figure 2.**
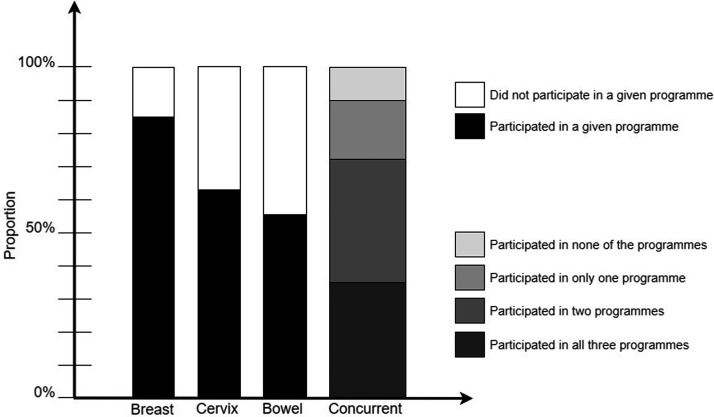
Participation in individual screening programmes, and concurrent participation in all three programmes in the last invitation round.

With completely random concurrent participation, 882 (29%) women would have participated in all three programmes, and 147 (5%) in none. The observed data showed that 1086 (35%) were screened for all three cancers, and 306 (10%) for none (Table 1). Hence, concurrent (non-) participation was not a random occurrence (*p* < 0.001 in both cases). Furthermore, 1142 (37%) women participated in two, and 526 (17%) in a single screening programme (Figure 2). Women who participated in a given screening programme were more likely to participate in both other recommended programmes than were women who did not participate in that programme (Table 2). Those who participated in that programme were less likely not to undergo screening in the other two programmes. For example, 43% of those who participated in breast screening also participated in both cervical and bowel screening, while only 11% did so among those who did not participate in breast screening. While 14% of those who participated in breast screening obtained no other cancer screening, 57% of those who did not participate in breast screening obtained no other cancer screening either. Nevertheless, only about half of participants in a given programme also participated in both other programmes (43%, 57%, and 66% of breast, cervical, and bowel screening participants, respectively).

**Table 1.** Individual-level data for 3060 English women: Concurrent participation in the three screening programmes, by screening programme.

Number of screening programmes attended	Participated in screening for			
	Breast cancer	Cervical cancer	Bowel cancer	N (%)
3	Yes	Yes	Yes	1086 (35)
2	Yes	Yes	No	639 (21)
	Yes	No	Yes	445 (15)
	No	Yes	Yes	58 (2)
1	Yes	No	No	355 (12)
	No	Yes	No	125 (4)
	No	No	Yes	46 (2)
0	No	No	No	306 (10)
Total	–	–	–	3060 (100)

**Table 2.** Individual-level data for 3060 English women: Concurrent participation in the three screening programmes, by whether the woman participated in a specific programme.

	Participated in additional screening programmes					
	Two		One		None	
Participated in screening for:	N (%)	RP (95% CI)	N (%)	RP (95% CI)	N (%)	RP (95% CI)
Breast cancer: no (N = 535)	58 (11)	1 (ref)	171 (32)	1 (ref)	306 (57)	1 (ref)
Breast cancer: yes (N = 2525)	1086 (43)	3.97 (3.10–5.08)	1084 (43)	1.34 (1.18–1.53)	355 (14)	0.25 (0.22–0.28)
Cervical cancer: no (N = 1152)	445 (39)	1 (ref)	401 (35)	1 (ref)	306 (27)	1 (ref)
Cervical cancer: yes (N = 1908)	1086 (57)	1.47 (1.36–1.60)	697 (37)	1.05 (0.95–1.16)	125 (7)	0.25 (0.20–0.30)
Bowel cancer: no (N = 1425)	639 (45)	1 (ref)	480 (34)	1 (ref)	306 (21)	1 (ref)
Bowel cancer: yes (N = 1635)	1086 (66)	1.48 (1.39–1.58)	503 (31)	0.91 (0.82–1.01)	46 (3)	0.13 (0.10–0.18)

CI: confidence interval; RP: relative proportion.

These results were reasonably robust for the cessation of screening due to hysterectomy and the gradual roll-out of bowel screening (not tabulated). Firstly, at age 60–65, 1725 (56%) women participated in both cervical and breast screening programmes, 983 (32%) participated in one, and 352 (12%) participated in none (Table 1). Before age 54, the overall picture remained similar: 1933 (63%) women participated in both, 874 (29%) in one, and 253 (8%) in neither of the two programmes. Secondly, among the 2391 women with an invitation to bowel screening sent in the year preceding their reference date or earlier, 988 (41%) attended all three screening programmes, 823 (34%) attended two, 361 (15%) attended one, and 219 (9%) attended none, compared with 35%, 37%, 17%, and 10%, respectively, in the main analysis that included all 3060 women (Table 1).

### General practice data

Of the 7014 practices, 3354 (48%) attained a breast screening coverage of at least 72.1%, 4075 (58%) attained a cervical screening coverage of at least 71.7%, and 3234 (46%) attained a bowel screening coverage of at least 57.3% (all three thresholds were national averages in 2017/18). In the most deprived areas (highest IMD tertile), only 5% of the practices attained above-average participation in all three screening programmes concurrently, whereas 62% of the practices attained below-average participation. An opposite pattern was seen among the practices with the lowest levels of deprivation, where 64% had consistently better screening coverage rates than the national average, and only 8% of the practices were underperforming. The ORs for deprivation and screening participation in the three programmes combined were highly statistically significant (Table 3). Similarly, practices with a higher proportion of unemployed patients and those with a higher proportion of smokers were less likely to attain a high coverage in all three screening programmes. Higher coverage was more frequent among practices with a higher proportion of carers, of individuals with long-term health conditions, and those with a high level of patient satisfaction with the practice itself. The patterns resembled a dose–response relationship, with the values for screening participation in the middle tertile of practice characteristics falling between those for the lowest and the highest tertiles, and those for the mixed-coverage practices falling between those for low-coverage and high-coverage practices.

**Table 3.** Data for 7014 English general practices: High-coverage practices and mixed-coverage practices compared with low-coverage practices, by practice population characteristics.

		Screening participation in all three programmes concurrently (row %)			OR for practices with mixed coverage vs. practices with below-average coverage		OR for practices with above-average coverage vs. practices with below-average coverage	
Non-screening indicators	Tertile (N = 7014)	Below average (N = 2288)	Mixed (N = 2345)	Above average (N = 2381)	Unadjusted (95% CI)	Adjusted^a^ (95% CI)	Unadjusted (95% CI)	Adjusted^a^(95% CI)
IMD score	Lowest (2314)	191 (8%)	645 (28%)	1478 (64%)	1 (ref)	1 (ref)	1 (ref)	1 (ref)
	Middle (2383)	658 (28%)	941 (39%)	784 (33%)	0.42 (0.35–0.51)	0.48 (0.38–0.60)	0.15 (0.13–0.18)	0.30 (0.23–0.40)
	Highest (2317)	1439 (62%)	759 (33%)	119 (5%)	0.16 (0.13–0.19)	0.23 (0.18–0.29)	0.01 (0.01–0.010	0.07 (0.05–0.10)
Unemployment	Lowest (2315)	249 (11%)	772 (33%)	1294 (56%)	1 (ref)	1 (ref)	1 (ref)	1 (ref)
	Middle (2383)	677 (28%)	887 (37%)	819 (34%)	0.42 (0.35–0.50)	0.55 (0.45–0.67)	0.23 (0.20–0.28)	0.44 (0.35–0.57)
	Highest (2316)	1362 (59%)	686 (30%)	268 (12%)	0.16 (0.14–0.19)	0.34 (0.28–0.41)	0.04 (0.03–0.05)	0.20 (0.15–0.26)
Long-term health conditions	Lowest (2315)	1249 (54%)	659 (28%)	407 (18%)	1 (ref)	1 (ref)	1 (ref)	1 (ref)
	Middle (2384)	585 (25%)	808 (34%)	991 (42%)	2.62 (2.27–3.02)	2.48 (2.10–2.94)	5.20 (4.47–6.05)	8.27 (6.36–10.74)
	Highest (2315)	454 (20%)	878 (38%)	983 (42%)	3.67 (3.16–4.25)	4.16 (3.47–5.00)	6.64 (5.67–7.78)	18.96 (14.01–25.66)
Carers	Lowest (2314)	1167 (50%)	704 (30%)	443 (19%)	1 (ref)	1 (ref)	1 (ref)	1 (ref)
	Middle (2388)	625 (26%)	845 (35%)	918 (38%)	2.24 (1.95–2.58)	1.85 (1.57–2.18)	3.87 (3.33–4.49)	3.25 (2.53–4.17)
	Highest (2312)	496 (21%)	796 (34%)	1020 (44%)	2.66 (2.30–3.08)	2.09 (1.76–2.49)	5.42 (4.65–6.31)	4.53 (3.47–5.91)
Smokers	Lowest (2313)	323 (14%)	542 (23%)	1448 (63%)	1 (ref)	1 (ref)	1 (ref)	1 (ref)
	Middle (2385)	681 (29%)	903 (38%)	801 (34%)	0.79 (0.67–0.94)	1.18 (0.94–1.48)	0.26 (0.22–0.31)	0.51 (0.39–0.68)
	Highest (2316)	1284 (55%)	900 (39%)	132 (6%)	0.42 (0.36–0.49)	0.85 (0.67–1.09)	0.02 (0.02–0.03)	0.08 (0.06–0.12)
Satisfaction with general practice	Lowest (2315)	1203 (52%)	719 (31%)	393 (17%)	1 (ref)	1 (ref)	1 (ref)	1 (ref)
	Middle (2386)	769 (32%)	888 (37%)	729 (31%)	1.93 (1.69–2.21)	1.63 (1.40–1.90)	2.90 (2.49–3.38)	1.73 (1.35–2.21)
	Highest (2313)	316 (14%)	738 (32%)	1259 (54%)	3.91 (3.33–4.59)	2.69 (2.24–3.22)	12.20 (10.31–14.42)	4.48 (3.45–5.82)

^a^Adjusted for all other practice characteristics in the table.

Note: Below-average participation: practices for which participation in all three screening programmes was below the national averages.

Mixed participation: practices in which participation in some screening programmes was below the national averages, and at or above the national averages in other programmes.

Above-average participation: practices attaining screening coverage in all three programmes at or above the national averages.

CI: confidence interval; IMD: index of multiple deprivation (the higher the score, the higher the level of multi-factor deprivation); OR: odds ratio.

## Discussion

To decrease the chances of dying from specific cancers, it is important for the population to attend all screening programmes as recommended. Virtually all women in our English dataset (98%) had ever undergone at least one cancer screening test. About four out of five women were up-to-date with breast, two out of three with cervical, and just over one in two with bowel screening. Although not entirely surprising given this variation in programme-specific participation, it is nevertheless disconcerting that only about one in three women were up-to-date with all their recommended cancer screens, and one in ten remained completely unscreened in the last round. While we observed the same patterns as elsewhere, in that participants in one programme were also substantially more likely to participate in another screening programme,^20,21,23,24,26^ only half of women who participated at least once did in fact participate in all other recommended screening. Taken together, this means that the targeted population is less well protected from all three cancers simultaneously than the participation rates from the individual screening programmes could suggest, although nine out of ten are protected against dying from specific cancers at least through one of the programmes.

Evaluations of cancer screening are typically undertaken for individual programmes. It may, however, be useful also to consider analysing the recommended combination of multiple programmes as a single package, recognizing that all cancer screening has a shared goal in avoiding premature mortality and reducing treatment complications. Also, while the individuals may prefer particular screening tests over others,^14^ they often do not have a nuanced perception of the differences between cancers.^35,36^ Studies such as ours provide some of the first indications of how the accumulation of multiple (evidence-based) screening programmes unfolds in practice. Our data clearly show that, for most women, screening does not appear to be a consistent habit, even when offered for free.

The detected deviation from the assumption of randomness would suggest that those who participate in all recommended screening may share certain traits, as do those who do not participate. Further exploration of these associations between multiple programmes could lead to a better understanding of the complexities in screening evaluation, such as self-selection. The latter can exaggerate the estimated benefit of screening on mortality.^26,37,38^ Participation is frequently thought to be more common among healthier individuals such as non-smokers, who would have better health outcomes even in the absence of screening.^20,26,39–43^ This was also suggested by the English general practice data. Nevertheless, the associations between population characteristics are probably more complex than they appear on the surface. For example, smokers are probably overrepresented among those who suffer from certain chronic conditions, and yet a high burden of chronic conditions within a general practice was associated with higher screening coverage rates in all three programmes combined. Being confronted with someone else’s disease and/or frailty as a carer also seemed to be positively associated with participation. It is possible that these patterns, rather than merely showing a direct association between patients’ health and cancer screening, also reflect the context of the frequency of contact with the general practice or health care in general, and/or the satisfaction therewith. Various other practice characteristics, such as deprivation and unemployment, also appeared to have a strong association with participation. While remaining mindful that these are ecological data, it appears that the wider social and economic context may play a role in motivating the population to undergo all recommended cancer screening.

These data still point to a role of personal characteristics that have been traditionally associated with (non-)participation in a single type of cancer screening,^10,14,15^ but the nature of this association requires further elucidation. While these factors may contribute to an individual opting for a certain type of screening, there seems to be an additional driver whereby the same person may find additional screening tests unacceptable, unnecessary, or simply not a priority. Future research may also need to investigate how the differences between the screening tests^14,25^ or factors related to an individual’s vulnerability^44,45^ affect participation in more than one screening programme. Finally, alternative venues for screening provision, for example one-stop screening clinics, could be considered, as well as alternative ways of approaching those women who obtain some but not all recommended screening.

Although the controls were age-matched to their cases, they were otherwise randomly selected from the invited population. Information on screening participation was obtained from national registers, minimizing the risk of selective recall, which may have been present in previous studies that tended to rely on self-reported data.^20–26^ In our study, the participation rates in each programme were similar to those reported in the official national statistics for all three programmes, suggesting that our data are representative for the invited population. The proportion who undergo all screening may these days be slightly lower, as screening participation has decreased by about three percentage points in each programme since 2010.^27,28,46^ Additional analyses showed that our conclusions were robust to the gradual roll-out of bowel screening and to cessation of screening due to hysterectomy, for which additional data were not available. Finally, our analysis would be further improved by having access to each woman’s sociodemographic background. Instead, we used general practice data from the same source population, although these ecological correlations should be interpreted cautiously.

## Conclusion

Despite satisfactory participation rates in the individual cancer screening programmes, only about a third of English women undergo all recommended cancer screening. Future studies should investigate the reasons why most of those who participate in some screening do not participate in all recommended screening, to inform policies which will contribute to fewer deaths from cancer.

## Data Availability

Fingertips data are publicly available on https://fingertips.phe.org.uk/. No additional unpublished data are available.
